# Prognostic Factors in Elderly Patients with Diffuse Large B-Cell Lymphoma and Their Treatment Results

**DOI:** 10.4274/tjh.galenos.2019.2018.0219

**Published:** 2019-05-03

**Authors:** Süleyman Cem Adıyaman, İnci Alacacıoğlu, Ayça Ersen Danyeli, Doğuş Türkyılmaz, Ömür Gökmen Sevindik, Fatih Demirkan, Özden Pişkin, Mehmet Ali Özcan, Bülent Ündar, Sermin Özkal, Güner Hayri Özsan

**Affiliations:** 1 Dokuz Eylül University Faculty of Medicine, Department of Internal Medicine, İzmir, Turkey; 2 Dokuz Eylül University Faculty of Medicine, Department of Hematology, İzmir, Turkey; 3 Dokuz Eylül University Faculty of Medicine, Department of Pathology, İzmir, Turkey

**Keywords:** Lymphoid cell neoplasms, B-Cell neoplasms, Lymphomas, Non-Hodgkin lymphoma

## Abstract

**Objective:**

Diffuse large B-cell lymphoma (DLBCL) is the most common type of non-Hodgkin lymphoma (NHL). The treatment of older NHL patients has always been a struggle; however, treatment statistics have begun showing favorable results similar to those of younger DLBCL patients thanks to newer treatment protocols. Here, we analyze the progress of our own elderly DLBCL patients who were followed between 2000 and 2016 in our center.

**Materials and Methods:**

Eighty-seven DLBCL patients, who were diagnosed and treated in the Dokuz Eylül University Department of Hematology between 2000 and 2016, were included in this study. Median age was 72 (65-89) years and 13 (14.9%) patients were older than 80 years.

**Results:**

Median follow-up time was 19 months and 45 patients (51.7%) died during the follow-up period. Median overall survival (OS) was 55 months and median progression-free survival was calculated as 27 months. Sixty-three patients (72.4%) received standard R-CHOP therapy. Complete response was seen in 46 (52.9%) patients. The median survival time for patients who had complete response was 136 months (p<0.001); however, OS was not statistically different between older (>80 years) and younger patients (p=0.236).

**Conclusion:**

According to our findings, we think that being able to complete standard R-CHOP therapy is vital for the survival rate of elderly DLBCL patients.

## Abstract (tr)

**Amaç:**

Diffüz büyük B hücreli lenfoma (DBBHL), Hodgkin dışı lenfomalar (NHL) arasında en sık görülen tiptir. Yaşlı NHL hastalarının tedavisi genellikle zorlu olmakla beraber günümüzde gelişen tedavi protokolleri ile birlikte yaşlı hastalarda da genç DBBHL hastalarına benzer oranda daha iyimser sonuçlar elde edilmektedir. Bu çalışmaya Dokuz Eylül Üniversitesi Hematoloji Anabilim Dalı’nda 2000-2016 yılları arasında takip ve tedavi edilmiş 87 yaşlı DBBHL hastası dahil edildi.

**Gereç ve Yöntemler:**

Yaş ortalaması 72 (65-89), 80 yaşının üzerinde 13 (%14,9) hasta mevcuttu. Ortalama takip süresi 19 ay olup, tüm takip süresinde hastaların 45’inin (%51,7) öldüğü gözlendi.

**Bulgular:**

Ortalama genel sağkalım süresi 55 ay iken progresyonsuz sağkalım süresi 27 ay olarak hesaplandı. Hastaların 63’ünün (%72,4) standart R-CHOP tedavisi almış olduğu görüldü. Ek olarak hastaların 46’sında (%52,9) tam yanıt alındığı gözlendi. Tam yanıt izlenen hastaların ortalama genel sağkalım süresi 136 ay olarak saptandı (p<0,001), ancak, 80 yaş üzeri hastalar ile altındaki hastaların genel sağkalım süreleri arasında anlamlı istatistiksel fark izlenmedi (p=0,236).

**Sonuç:**

Bu veriler ışığında yaşlı DBBHL’li hastalarda standart R-CHOP tedavisinin tamamlanmasının sağkalım süresine önemli etkisi bulunduğunu söyleyebiliriz.

## Introduction

Diffuse large B-cell lymphoma (DLBCL) is the most common non-Hodgkin lymphoma (NHL) type and accounts for 30%-40% of all NHL cases [1]. In the United States, its incidence is 7/100,000 [2], whereas in Europe it is 4.9/100,000 [3]. In Turkey, based on 2014 data, the incidence of DLBCL is 6/100,000 [4]. The diagnosis rate for DLBCL is higher in Caucasians than Africans and Asians [5]. The median age of DLBCL patients is 64 years and it is slightly more common in men than women (male/female=1.5) [2].

According to recent studies, approximately 70% of all newly diagnosed neoplasms will be observed in geriatric patients by 2020 [6]. Nearly 53% of all the newly diagnosed NHL patients are over 65 years old [7]. Because of their comorbid diseases and their frail conditions, elderly patients have always been considered poor responders to treatment in comparison to younger patients. However, recent studies with newer treatment agents have shown that older DLBCL patients may also respond well to treatment, like younger patients. According to early studies conducted with elderly DLBCL patients when the CHOP regimen alone was the standard of care, the rate of complete response (CR) was 40% to 50% and 3-year overall survival (OS) was around 30%, which was considered unsatisfactory, whereas in studies conducted in the rituximab era, CR was reported as 60% to 80% and 3-year overall survival rates were found to be around 70% [8]. 

In light of this information, it is especially important to consider the right treatment option for elderly DLBCL patients. In this study, we analyzed the progress of our own elderly DLBCL patients who were followed between 2000 and 2016 in our center.

## Materials and Methods

A total of 87 patients were included in this study, all of whom were diagnosed and followed between 2000 and 2016 in the Dokuz Eylül University Hospital Department of Hematology. Consent of the patients and approval were obtained from Dokuz Eylül University Ethics Board (date: 28.1.2016 2425-GOA, approval number: 2016/03-24) before the start of the study. Patients who had previously been diagnosed with another form of malignancy were excluded from the study. In this retrospective study, university archives were used to analyze patient information such as age, time of diagnosis, stage, treatment regimes, treatment results, side effects, and comorbid diseases. Additionally, in order to assess the pathological subtype of DLBCL, immunohistochemical (IH) staining was applied to some of our patients’ biopsy materials by the department of pathology with the informed consent of patients and/or their relatives.

### Immunohistochemical Method

IH stains were applied to some patients’ biopsy materials whose subtypes were unknown. To achieve that, formalin-fixed, paraffin-embedded sections of 4 µm were collected from patient samples. On lysine slides, CD10 (VENTANA anti-CD-10 SP67 1:100 dilution), Bcl-6 (Cell Marque, 1:200 dilution), and MUM-1 (VENTANA, 1:100 dilution) stains were applied. Afterward, these slides were analyzed under a microscope.

### Statistical Analysis

Assessment of the acquired data was done with SPSS 17. The suitability of the numeric variables was assessed using the Kolmogorov-Smirnov test. The chi-square test was used to test the relationship between two categorical variables. Kaplan-Meier analysis was used in order to show the impact of prognostic factors on survival rates. For multivariate data analysis, the Cox regression method was used. Probability values less than 0.05 were considered statistically significant.

## Results

The median age of patients was 72 (65-89) years. Forty-seven patients (54%) were male and 40 (46%) female. Seventy-four (85.1%) of our patients were younger than 80 years old and 13 (14.9%) of them were older than 80. Median follow-up time was 19 (1-180) months. In total, 45 patients (51.7%) died during follow-up. Comorbid diseases such as type 2 diabetes, hypertension, and congestive heart failure were seen in 62 of our patients (71.3%), whereas 25 (28.7%) patient had no previously diagnosed comorbidities (Table 1).

Among the 51 patients whose subtypes could be identified by immunochemical staining, 14 (27.5%) were classified as having the GCB (germinal-center type B-cell) subtype and 37 (72.5%) as non-GCB. Patients were also categorized using Ann Arbor staging: 11 (12.6%) patients as stage 1, 31 (35.6%) as stage 2, 22 (25.3%) as stage 3, and 23 (26.4) as stage 4. We also calculated patients’ International Prognostic Index (IPI) scores. Patients with IPI scores of 2 and 3 were in the majority with 30 (34.5%) patients each, while there were 15 (17.2%) patients with an IPI score of 4, 12 (13.8%) patients with an IPI score of 1, and no patients with an IPI score of 5 (Table 2).

Most of the patients (72.4%) were treated with R-CHOP at regular dosage, 2 patients (2.3%) with reduced-dose R-CHOP, 4 patients (4.6%) with R-CEOP, 4 patients (4.6%) with R-CVP, 10 patients (11.5%) with CHOP, 1 patient (1.1%) with mini-CEOP, and 3 patients (3.4%) with R-steroid. Anthracycline-based chemotherapy was given to 75 patients (86.2%) in total, whereas rituximab was used for 76 (87.4%) of all patients. Additionally, high-risk patients received the proper intrathecal methotrexate as part of the CNS prophylaxis strategy according to their National Comprehensive Cancer Network central nervous system risk score. Radiotherapy was used in 24 (27.6%) patients (Table 3).

Sixty patients (69%) were able to complete their designated therapy and, among those patients, 46 (52.9%) had CR. When comparing IH subtypes and treatment results, we saw that 9 (64.3%) patients with the GCB subtype had CR, whereas in patients with non-GCB subtypes, 14 (37.8%) had CR. However, statistically we found no significant difference between the IH subtypes (p=0.174). CR was seen in 6 patients (46.2%) who were older than 80 and in 40 patients (46%) who were under the age of 80; there was no statistically significant difference between the treatment results and age (p=0.585).

The most common side effect in this study was neutropenia, which occurred in 65 (74.7%) of our patients. Other side effects were heart failure, neuropathy, pneumonia, sepsis, renal failure, thrombosis, and reactivation of tuberculosis.

**Figure 1:**
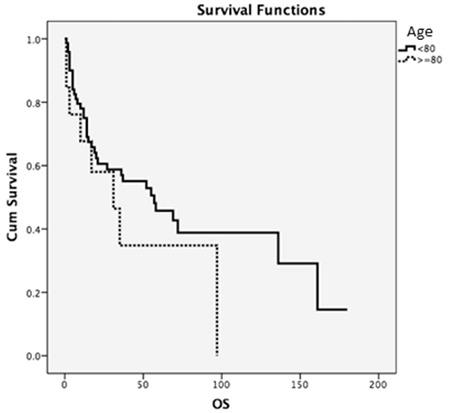
Overall survival and age. OS: Overall survival.

Median progression-free survival time (PFS) was 27 months. Median OS time in our study was 55 months, while 3-year, 5-year, and 10-year OS was calculated as 54%, 44%, and 33%, respectively. For patients older than 80 years old, OS was 31 months, while in younger patients it was 57 months. However, no significant statistical difference was found between the two groups (p=0.236) (Figure 1).

**Figure 2:**
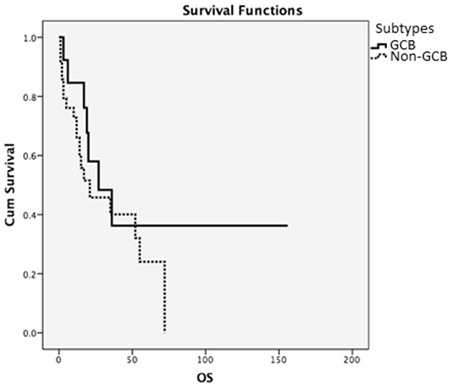
Overall survival and immunohistochemical subtypes. OS: Overall survival.

OS in patients with the GCB subtype was 27 months and in patients with non-GCB subtype the OS was 21 months; between these two subtypes we found no statistically significant difference (p=0.218) (Figure 2). In patients who could complete standard-dose R-CHOP therapy, 3-year OS was 48% for the GCB group and 42% for non-GCB.

Even though we found no statistical difference between stage and OS (p=0.999), we did find a statistical difference between IPI scores and survival rates. Among patients whose IPI score was 1, median survival time was 97 months, and in those who had an IPI score of 4, it was 14 months (p=0.008). When the general survival rate was evaluated in our elderly patient group, IPI score was detected as an independent predictive feature even if it was adapted using sex and comorbidity in Cox regression analysis (p=0.003).

**Figure 3:**
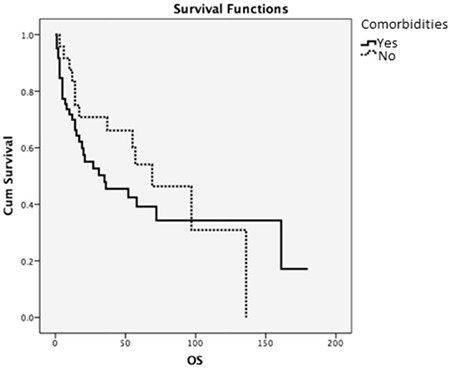
Overall survival and comorbid diseases. OS: Overall survival.

The median survival time was 69 months for patients who had no previous comorbid disease and 35 months in patients who had previously been diagnosed with a chronic comorbid disease; statistically, there was no significant difference (p=0.366). The 3-year OS was calculated as 70% in patients without comorbidities and 45% for those who had an accompanying chronic disease (Figure 3).

The median survival rate for patients who received rituximab was 58 months and 3-year OS was 57%, whereas for patients who did not receive rituximab, the median survival rate was 27 months and 3-year OS was found as 36% (p=0.379). Additionally, 5-year OS and 10-year OS for patients who received rituximab therapy was 47% and 37%, respectively.

**Figure 4:**
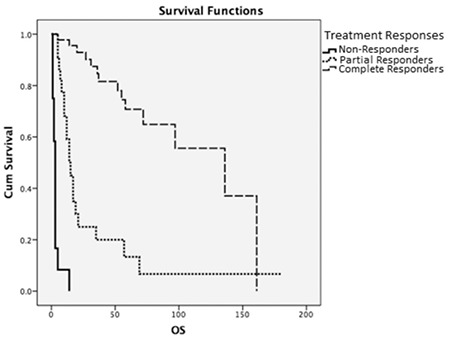
Overall survival and treatment response. OS: Overall survival.

The median survival of patients who had a complete treatment response was 136 months, which was statistically significant compared to other patients who had partial response or non-responders (p<0.001) (Figure 4).

Relapse was seen in 22 patients (25.3%). Median relapse time was 16.5 (3-132) months. The OS in patients with an early relapse (<1 year) was 14 months, whereas it was 69 months for those with late relapse (>1 year), which was statistically significant (p=0.025). The PFS in patients with early relapse (<1 year) was 8 months, whereas it was 27 months for those with late relapse (>1 year), which was also statistically significant (p<0.001).

## Discussion

DLBCL is the most common type of NHL and its prevalence grows with age [1]. Treatment options have always been a struggle in elderly patients for reasons like frailty and comorbid diseases [9]. According to other previous studies, being unable to receive appropriate treatment, the standard treatment regimen being R-CHOP, usually has negative effects in elderly patients [10]. DLBCL is an aggressive hematologic malignancy and patients diagnosed with DLBCL have an average lifespan of less than 1 year without treatment. However, as recent studies show, with newer and improved therapy options the survival rates are much better than before in older DLBCL patients [11].

In our study, we analyzed DLBCL patients who were older than 65 years retrospectively. Our population was between the age of 65 and 89 (mean: 72) years and the male/female ratio was 1.17, which was similar to the literature [2]. Additionally, we studied IH subgroups based on the Hans algorithm [12]. In various other studies, it has been shown that non-GCB subtypes are more common in older DLBCL patients. In 51 patients whose subgroups we could identified, we found that most of them (72.5%) were patients with non-GCB subtype, which was similar to the literature [13]. Based on the literature, 60% of patients diagnosed with DLBCL who are older than 70 have an accompanying chronic disease [14], while in our study group 62 patients (71.3%) had other comorbidities and the most common comorbid disease was diabetes mellitus type 2 (17.2%), followed by hypertension (14.9%) and heart failure (14.9%), respectively. In our study group, patients mostly received standard-dose R-CHOP therapy (72.4%). In the literature, neutropenic fever is the most common side effect seen in DLBCL patients, and in elderly patients its frequency is nearly 40% [15]. Consistent with other studies, neutropenic fever was the most common side effect (42.5%) in our study.

In this study, the treatment completion rate in general was 69% and 52.9% of the patients had complete treatment response. Furthermore, CR was higher (76.1%) in patients who received R-CHOP therapy, which was similar to other larger randomized studies such as RICOVER-60 [11]. Additionally, we saw a 64.3% CR rate in patients with the GCB subtype, whereas 37.8% patients with non-GCB had CR. According to other studies in the past, patients with GCB subtypes were found to be better responders to treatment compared to patients with non-GCB subtypes; however, according to recent studies conducted after the rituximab era, the two subtypes have begun showing similar treatment results. A 10-year prospective study conducted in Finland with 194 DLBCL patients showed that adding rituximab to therapy leads to better survival rates in patients with non-GCB subtypes [16]. Though it seems that there was a favorable outcome in our study for patients with the GCB subtype, no statistically significant difference was discovered (p=0.205). This may be explained by the small number of patients and the fact that we included patients from both the pre-rituximab and post-rituximab era in our study population. There are many studies in the literature that analyzed the relationship between age and treatment results. A large retrospective study conducted by Thieblemont et al. [17] after the rituximab era in 2008 revealed that even though younger patients showed more favorable results, there were no statistically significant differences between younger patients and patients who were older than 80. We also found similar results within our study group; patients older than 80 had 46.2% CR whereas younger patients had 46% CR (p=0.572).

In our study, median survival time in general was calculated as 55 months. We also found that, for patients younger than 80 years old, overall median survival time was 57 months, whereas for older patients it was 31 months. However, there were no statistically significant differences between the two groups (p=0.236). In several large studies of DLBCL patients, in the German DLBCL group of Pfreundschuh et al. [11], in patients between the ages of 60 and 80 who received rituximab, the 3-year OS was found as 78%. In another large-scale study conducted by the GELA group, the 10-year OS was shown as 44% for patients who received rituximab treatment [18]. Among the patients receiving rituximab in our study, the 3-year OS was calculated as 54% and 10-year OS was 37%. For patients older than 80 years, the 3-year OS was 38%, and for those younger than 80 it was 58%. The difference between the literature and our study might be explained by the different current lifespans of those countries (France, Germany, etc.) and ours. According to 2015 WHO data, life expectancy in Turkey is 75.8 years, while it is 82.5 in France and 81.1 in Germany. Additionally, in our study population, we had patients older than 80 years old and that was also different from those larger studies in which the maximum age was 80.

Even though we could not identify all of our patients’ subgroups, we found that median survival time was 27 months for GCB types and 21 months for patients with non-GCB subtypes, for which there was not a significant statistical difference (p=0.218). In a large study of DLBCL patients between the ages of 23 and 88 conducted by Seki et al. [19], 3-year OS was found as 68% in patients with the GCB subtype and 67% for non-GCB among patients who received R-CHOP. In our study, we found that the 3-year OS of patients who received R-CHOP with GCB subtype was 48% while it was 42% for non-GCB subtypes, which was consistent with the literature of the post-rituximab era. When we analyzed the effect of disease stage on patients, we found no statistical difference; median survival time of patients with stage 1 was found as 72 months and that of stage 4 was calculated as 69 months (p=0.999). However, like other previous studies [20], we found a statistically significant difference regarding the IPI scores of our patients. The median survival time was 97 months for patients with IPI scores of 1 and 14 months for patients with IPI scores of 4 (p=0.008). In the literature, there are studies that show the negative effects of comorbidities on elderly DLBCL patients [14]. In our study, the median survival time for patients without any comorbid diseases was found as 69 months, whereas it was 35 months for patients with an accompanying chronic disease; however, though the survival times of the patients without comorbidities seemed favorable, there was not a statistically significant difference (p=0.366).

Before the age of rituximab, anthracycline-based CHOP therapy was considered the standard therapy for patients with DLBCL and the cure rates in those times were around 50%-60% for younger patients and 25%-30% for older patients [21,22,23]. In the GELA group study of Coiffier et al. [18], 10-year OS for the patients who only received CHOP therapy was 28% and they showed that adding rituximab to therapy increased the 10-year OS to 44%. Among our study group, the median survival time of patients receiving rituximab was calculated as 57 months, compared to 27 months for those who did not receive rituximab treatment. While we saw a tendency in favor of the rituximab group, it was not statistically significant (p=0.513). Despite old age and frailty, 60 (69%) of our patients could complete their designated therapies and 63 (72.4%) of them received full standard-dose R-CHOP therapy. Among those patients, we saw a complete treatment response in 46 (52.9%), among whom the median survival time was calculated as 136 months, and that was statistically significant (p<0.001). The median relapse time in our study was calculated as 16.5 months and the relapse rate was 25.3%. In other studies conducted among similarly aged patients (60-80 years), relapse rates were found to be generally higher (up to 51%) [8]. This difference might be explained by the fact that our study group was relatively smaller. However, similar to the literature, we did find a statistically significant difference between early relapses (<1 year) and late relapses (OS, p=0.025; PFS, p<0.001).

## Conclusion

We think that in elderly DLBCL patients, being able to complete the rituximab-based standard therapy regimen directly affects survival rates. Additionally, comorbidities and higher IPI scores have very important effects on the survival of DLBCL patients. Based on our results, we can safely say that treating elderly DLBCL patients with standard R-CHOP therapy is very important and shows favorable results similar to those of younger patients.

## Figures and Tables

**Table 1:**
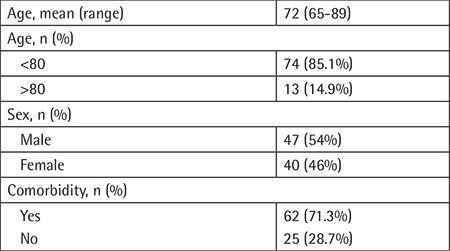
Patient demographics and clinical data.

**Table 2:**
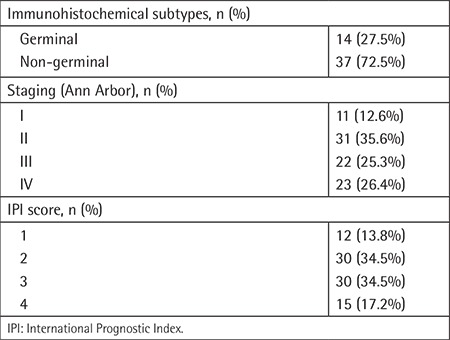
Tumor-related characteristics.

**Table 3:**
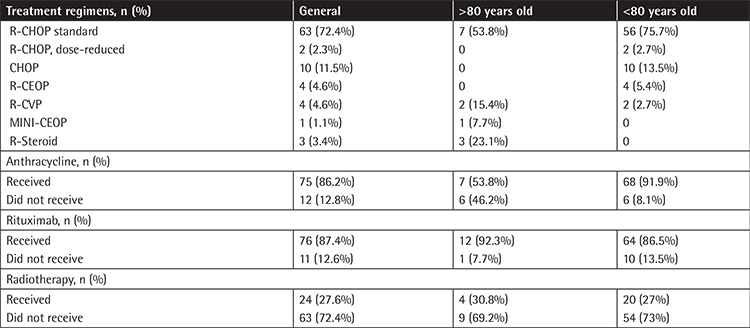
Patient treatment statistics.
